# The Lived Experience of Couples Undergoing In Vitro Fertilisation in Greece: An Interpretative Phenomenological Analysis

**DOI:** 10.3390/healthcare14060802

**Published:** 2026-03-21

**Authors:** George Koulierakis, Apostolia-Konstantina Theodosiou, Eleftheria Karampli, Angeliki Liarigkovinou

**Affiliations:** 1 Laboratory of Epidemiology, Health Determinants and Well-Being, Department of Public Health Policy, University of West Attica, 11521 Athens, Greece; apostheo1994@gmail.com; 2 Laboratory for Health Technology Assessment, Department of Public Health Policy, University of West Attica, 11521 Athens, Greece; ekarabli@uniwa.gr (E.K.); aliarig@uniwa.gr (A.L.)

**Keywords:** in vitro fertilisation, medically assisted reproduction, infertility, couples’ lived experience, psychological impact, interpretative phenomenological analysis, qualitative study, Greece

## Abstract

**Background/Objectives**: Research examining the emotional and psychological challenges experienced by couples undergoing in vitro fertilisation (IVF) remains limited. Existing evidence suggests that women undergoing IVF often report elevated levels of depression, anxiety, and emotional distress, whereas men may experience feelings of anger, inadequacy, and self-doubt, especially following unsuccessful treatment cycles. Successful IVF outcomes are commonly associated with intense joy, relief, and fulfilment as couples realise their aspiration to become parents. In light of the limited qualitative research conducted in Greece to date, in the present study, we aimed to explore the lived experiences of couples undergoing IVF treatment, with particular attention to emotional, relational, and systemic dimensions. **Methods**: A qualitative research design was employed. Semi-structured, in-depth interviews were conducted with six heterosexual couples (aged 18–49 years) residing in Athens and Karditsa, Greece, all of whom had undergone IVF treatment. Interviews were audio-recorded, transcribed verbatim, and analysed using Interpretative Phenomenological Analysis. **Results**: Our analysis revealed five interrelated superordinate themes with associated subordinate themes: (1) making sense of infertility and IVF, (2) negotiating relationships under the strain of IVF, (3) IVF as an emotionally demanding journey, (4) navigating institutional and systemic barriers, and (5) projecting the future through IVF experience. Lived experiences of infertile couples undergoing IVF treatment highlighted a range of emotions, social pressure, and attitudes towards IVF and related policies. **Conclusions**: In Greece, where demographic decline has been widely discussed in policy debates, IVF has gained societal and policy attention. For many participants, IVF represented a hopeful pathway towards achieving parenthood despite the emotional and practical challenges involved.

## 1. Introduction

### 1.1. Infertility and the Growing Use of Assisted Reproductive Technologies

For many couples, the process of conceiving is straightforward; for others, however, attempts to conceive can become prolonged and emotionally demanding. The majority of healthy young couples (85–90%) conceive within the first 12 months [1]. When conception does not occur after one year of regular attempts, couples may face infertility [2]. Infertility is commonly defined as the inability to conceive or carry a pregnancy to term naturally [3]. Recent estimates suggest that infertility affects around 17% of the global adult population, making it a significant public health concern [4].

Beyond its biomedical definition, infertility is a complex life experience that may affect individuals’ psychological well-being, social identity, and relationship dynamics [5,6]. The inability to conceive can challenge deeply embedded social expectations surrounding parenthood, family formation, and gender roles. In many societies, becoming a parent is considered an important life milestone, and difficulties achieving pregnancy may therefore be associated with feelings of inadequacy, stigma, and emotional distress [7].

To address infertility, many couples turn to medically assisted reproduction (MAR), with in vitro fertilisation (IVF) being the most common procedure [8]. IVF aims to enhance fertility or prevent genetic disorders [9,10], and while it has high success rates [11], it also entails long-term emotional, economic, and social challenges [12,13]. IVF success depends on factors such as maternal age, reproductive history, infertility causes, lifestyle, and various clinical factors [14,15,16,17]. These uncertainties can contribute to emotional strain for individuals and couples navigating fertility treatment.

### 1.2. Psychosocial Dimensions of Infertility and IVF

A growing body of research has examined the psychological and social consequences of infertility and fertility treatment. In quantitative studies, researchers have consistently documented elevated levels of stress, anxiety, and depression [18,19]. Individually, women bear a greater psychological burden [20], lack of support, and feelings of isolation [21], partly due to the physical demands of treatment and the social expectations surrounding motherhood [22]. Men, although less frequently studied, may also experience emotional distress related to infertility diagnoses, financial pressures, and self-doubt [23].

Beyond individual psychological outcomes, infertility and IVF may influence couples’ relationships and social interactions. Couples must navigate negative societal reactions towards infertility and infertility-related stigma [24], affecting their emotional well-being and social interactions [25], while assisted reproductive technology (ART)-related pregnancies challenge couples’ social and relational bonds [26].

In previous studies researchers have also highlighted the issue of access to treatment and equity in reproductive healthcare [27]. The financial costs associated with IVF can be substantial, raising questions of access, equity, and support [28,29] and highlighting the need for better insurance coverage and financial support [30,31].

### 1.3. Qualitative Perspectives on Infertility Experiences

In qualitative research, infertility has been described as a form of biographical disruption, challenging individuals’ sense of identity and life trajectory [7], and the emotional complexity of IVF treatment has been highlighted, often characterised as an “emotional rollercoaster” [32]. Furthermore, qualitative studies conducted in different cultural contexts have shown that infertility experiences are shaped by social norms surrounding gender, family, and reproduction [7] and that couples negotiate different aspects of the IVF procedure, such as treatment decisions, emotional responses, and social support [33,34].

### 1.4. Theoretical and Methodological Framework: Interpretative Phenomenological Analysis

In order to explore couples’ experiences of IVF in depth, in the present study, we adopted Interpretative Phenomenological Analysis (IPA), a qualitative research methodology concerned with examining how individuals make sense of significant life experiences [35,36,37]. IPA was selected because it enables an in-depth exploration of how participants make sense of emotionally complex and relationally embedded experiences such as IVF [38]. IPA was uniquely suited to our aim of capturing the depth, nuance, and subjectivity of couples’ lived experiences. Its idiographic emphasis enabled us to explore both individual and dyadic meaning-making, while its interpretative dimension acknowledged the co-construction of meaning between participants and researcher.

### 1.5. Rationale for a Dyadic Approach

Overall, research focusing on couples as a unit remains limited, particularly in Greece. Yet infertility is rarely an individual phenomenon: it is inherently relational, shaping and being shaped by the couple’s shared experiences, decision-making, and emotional coping strategies [39]. Adopting a dyadic approach enables us to capture how IVF influences both partners and their relationship, highlighting dynamics such as spousal support, resilience, and the negotiation of future parenthood [40]. This approach is especially relevant in cultural contexts, such as Greece, where fertility has traditionally been closely tied to marriage, with births outside marriage remaining relatively rare compared with many other European countries [41].

### 1.6. Study Aims

To the best of our knowledge, no study investigating the lived experiences of couples undergoing IVF treatment in Greece has been conducted to date. Thus, we seek to address this gap by posing the following research questions:

(a) How do couples in Greece experience the psychological and emotional challenges of undergoing IVF?
(b) How does IVF impact couples’ relationships, both within the partnership and with their wider social environment?
(c) How do couples perceive the role of the healthcare and insurance systems in shaping their IVF experiences?
(d) What expectations and meanings do couples attach to their future after undergoing IVF?

## 2. Materials and Methods

### 2.1. Recruitment and Participants

Six couples who had previously undergone IVF, regardless of the treatment outcome, were purposively recruited through a private fertility clinic in Athens and the first author’s professional network in Karditsa (Table 1). Inclusion criteria required participants to be aged 18 years or older, reside in Greece, and have undergone at least one cycle of IVF. Exclusion criteria included being under 18 or living outside Greece. Participation by both partners was a strict requirement. If either partner was unwilling to participate, the couple was excluded from the study. Thus, eligibility for the survey was limited exclusively to couples in which both partners agreed to take part. This recruitment strategy enabled access to individuals with direct experience of IVF treatment, yet it may have introduced selection bias, potentially favouring participants who were more treatment-engaged or had relatively stable relationships with fertility services.

The decision to include six couples was based on the principles of Interpretative Phenomenological Analysis (IPA) [27], which recommends small, relatively homogenous samples to enable detailed, idiographic exploration of lived experience. This sample size is consistent with prior IPA studies, which typically include between 4 and 10 cases [35,42].

Recruitment took place through two channels. In Karditsa, local doctors invited eligible couples to participate and then forwarded their contact information to the first author. In Athens, recruitment occurred via a psychologist at the collaborating fertility clinic, who referred interested couples to the first author. All participants received full study information and provided written consent before participation. None of the participants refused to participate or withdrew after they had given consent.

### 2.2. Data Collection and Saturation

Data were collected through semi-structured, face-to-face interviews using an interview guide (Supplementary Document S1) covering three areas: (1) experiences before undergoing IVF, (2) the IVF process, and (3) life after IVF. Additional probing questions were asked when needed. Interviews, conducted by the first author between October and December 2023, were digitally recorded, anonymised, and transcribed verbatim by the second author (female, economist, MSc). The first author reviewed the transcripts for accuracy. Νο field notes were taken following each interview. There was no prior relationship between the interviewer and participants, and none declined to participate or withdrew at any stage of the study. Transcripts were not returned to participants for revisions, and no follow-up interviews were conducted. Interviews lasted approximately 25 min on average. While the interviews were shorter than in some IPA studies, the relatively focused interview duration was sufficient to elicit narratives addressing the research questions.

Partners were interviewed jointly, as infertility and IVF are inherently relational experiences and we aimed to capture the shared meaning-making processes of couples as they negotiated treatment and parenthood together. Nevertheless, joint interviews might also shape the narratives produced. As both partners were present, the construction of collaborative accounts might be encouraged, while the expression of conflict, disagreement, or sensitive experiences might be constrained. The interviewer intervened when necessary to mitigate this limitation, manage intra-dyadic divergences in lived experience, and address potential power asymmetries during joint narration. Divergent partner experiences were addressed analytically by representing both individuals’ perspectives. Relational dynamics were preserved and not subsumed under generalised themes.

Data collection continued until the narratives provided sufficient analytic depth to address the research questions. Consistent with Braun and Clarke’s (2019) critique of formal “data saturation” [43], the aim was not to exhaust perspectives, but to develop a rich understanding of couples’ meaning-making. The dataset was therefore considered adequate to illustrate participants’ lived experiences.

Interviews were held in a private space in the clinic, cafés, or homes to ensure a comfortable, secure environment for couples to share their experiences. In cases in which couples explicitly requested cafés as the location to be interviewed, as these environments felt more comfortable and less intimidating, interviews were conducted in quiet, secluded areas to minimise the risk of being overheard.

Couples were fully informed about the study and provided written consent to participate. Before the interviews, an informal conversation established rapport, during which the interviewer explained the study’s objectives, the interview process, and participants’ rights, including the right to withdraw at any time. Participants could ask questions before signing the consent form. They did not provide any feedback on the results.

### 2.3. Researchers’ Reflexivity

The first author, a male health psychologist with experience in qualitative health research and IPA, supervised the study design and implementation. His professional background informed both the interview framework and the interpretative approach to participants’ narratives. The second author, a female economist with postgraduate training in public health research, conducted the interviews. Her interest in infertility, IVF, and related health policy issues informed her engagement with participants. During the interviews, she adopted a respectful and empathetic stance towards participants while remaining attentive to the potential influence of her own perspectives on the research process. The analytic process involved regular discussions between the first and second authors, during which interpretations were critically examined and refined. This collaborative reflection helped challenge individual assumptions and enhanced the credibility of the analysis.

### 2.4. Ethical Considerations

Ethical approval was granted by the Research Ethics Committee of the University of West Attica (Approval No 72553-28/07/2023) and the Bioethics Committee of the collaborating clinic (Approval No 203–27/7/2023).

### 2.5. Analysis

The transcribed interviews were pseudonymised to ensure confidentiality and analysed using Interpretative Phenomenological Analysis (IPA) [36,37]. The second author conducted the analysis, without the use of any software in three iterative stages [44]. Each transcript was read repeatedly to enable immersion in the data and generate descriptive, linguistic, and conceptual notes. Emergent themes were then developed by examining how participants made sense of their experiences. Themes within each case were examined for conceptual connections, clustered into subordinate themes, and organised into superordinate themes consistent with IPA’s idiographic commitment. Each couple’s account was analysed in depth before cross-case comparisons were conducted. Throughout the process, interpretations were continually checked against the original transcripts to ensure that themes remained grounded in participants’ accounts.

The methodology and reporting of findings follow the COREQ guidelines [45] (Supplementary Table S1).

### 2.6. Trustworthiness

The study’s quality was assessed using Yardley’s (2017) [46] principles for evaluating qualitative research. To ensure that the analysis remained grounded in the participants’ accounts, the second author engaged in repeated, detailed readings of each transcript, before developing higher-level interpretations. Emergent themes were developed inductively and consistently cross-checked against verbatim extracts to ensure close alignment with participants’ narratives. Reflexive discussions between the first and second authors took place throughout the analytic process to review interpretations and refine the clustering of themes. In keeping with IPA’s idiographic commitment, each couple’s account was analysed in depth as a standalone case before cross-case comparisons were undertaken, enabling both shared experiential patterns and important divergences between couples to emerge. Transparency was further supported through a clear presentation of the analytic process and illustrative participant quotations.

## 3. Results

Five interrelated superordinate themes, including subordinate themes, emerged: (1) making sense of infertility and IVF, (2) negotiating relationships under the strain of IVF, (3) IVF as an emotionally demanding journey, (4) navigating institutional and systemic barriers, and (5) projecting the future through IVF experience (Supplementary Table S2).

### 3.1. Making Sense of Infertility and IVF

This theme reflects how participants interpret and position themselves in relation to infertility and IVF through their attitudes and experiences during their journey in seeking medical assistance and consulting with appropriate healthcare professionals.

#### 3.1.1. Experiencing Infertility

Infertility was experienced as a deeply disruptive event that challenged couples’ expectations to become parents. Couples’ journey started with the medical diagnosis. Couples described feelings of helplessness as they attempted to make sense of it and accept infertility.

*They said that we were not capable of having children. … that my wife’s egg cells were no good and that my own sperm was also not good at all.*

(Menelaos)

#### 3.1.2. Seeking Help for IVF

Taking the decision to undergo IVF was framed as a necessary step towards restoring the possibility of parenthood, representing couples’ active effort to regain control over their infertility.

*We needed help. And then we decided to undergo IVF.*

(Julia)

*It can be done, all of this, and we started the process here with my gynecologist. He sent me to [she mentions a big city] to a private clinic, to a fantastic doctor—really, I can’t say otherwise. He was one of the few doctors we trusted, and we decided that yes, we had to do it.*

(Thetis)

#### 3.1.3. Making Sense of IVF

Some couples felt that people’s perceptions were influenced by their own experiences, shaping positive or negative opinions, yet they noted a shift in attitudes towards IVF, from social stigma to viewing it as a path to parenthood.

*It’s better now…. Nowadays, people say: ‘Oh, are you going to have IVF. Congrats’.*

(Thetis)

*“Nowadays… because there is so much infertility, people ask you what IVF is so that they can do it themselves as well.”*

(Hercules)

### 3.2. Negotiating Relationships Under the Strain of IVF

This theme explores how IVF reshapes couples’ marital and social relationships, including the reactions of significant others to their decision to undergo IVF.

#### 3.2.1. Strengthening the Spousal and Parental Bond

For many couples, IVF strengthened emotional solidarity within their relationship. Participants reported a strong spousal bond marked by love, support, and composure, rather than conflict. When treatment resulted in a child, the strong parent–child bond was often experienced as particularly meaningful.

*During my difficult times, my husband was there to support me.*

(Julia)

*We have a strong bond with our daughter. I feel like a normal mother, not overprotective, raising her like my mother raised me.*

(Marcella)

Nevertheless, one participant’s discourse reflected the fear of straining the spousal bond and the vulnerability IVF can trigger within the couple dynamic.

*It was my fear that if we did not have children, we would lead ourselves straight forward to divorce.*

(Marcella)

#### 3.2.2. Negotiating Disclosure and Social Reactions to IVF

Couples carefully negotiated disclosure of their IVF journey. Some openly shared their decision to pursue IVF, while others confided in only trusted individuals and a few kept the decision private. Reactions from the social environment included support, understanding, criticism, and fears.

*We didn’t hide our IVF decision…. Some people were very supportive and understood our desire to become parents*.

(Marcella)

*He had a colleague… and his wife said in front of everyone: ‘You won’t conceive… you will have to do IVF.’ It’s not polite to say something like that in front of someone*.

(Hyacinth)

### 3.3. IVF as an Emotionally Demanding Journey

This theme covers couples’ IVF experiences, including challenges such as miscarriages, injections, anaesthesia, and egg retrieval, emotional impacts, such as distress and anxiety, and existential dimensions of undergoing the procedure.

#### 3.3.1. Enduring the Emotional and Physical Challenges of IVF

Participants described the IVF procedure as emotionally and physically demanding. The preparation phase, the intake of medication, and the egg retrieval procedure with anaesthesia were particularly distressing for women, placing them under considerable mental strain. Miscarriage and failed attempts were experienced as sources of loss, sadness, and emotional breakdowns.

*… We lived that pregnancy till the sixth week… It was better for me to know that there is no baby, 12 days after our IVF attempt, rather than to live this bad experience again…*

(Calliope)

When IVF embryo conception failed, couples’ narratives revealed a divergence in emotional response, suggesting differences in expectations and coping processes within the dyad.

*During our first unsuccessful IVF attempt, Archelaus was emotionally devastated, I did not. Prior to this experience, it was my firm belief that if an experienced IVF centre took the IVF’s responsibility… I thought that IVF was an easier procedure. In fact, IVF was more difficult than I thought…*

(Hyacinth)

Women experienced the physical demands of treatment most intensely, while men faced stress, fear, and anxiety about both their partner’s health and the outcome. At times, women had to support their husbands through the process.

*My main anxiety was to see my Hyacinth getting out completely well of her surgery. That was my first and basic anxiety.*

(Archelaus)

#### 3.3.2. Resilience and Imposter Syndrome

Resilience emerged as an important coping strategy stemming from a “never give up” mentality, positive thinking, and acceptance of life’s challenges. Some required a mental health break to strengthen their resilience, while others struggled with disappointment and feelings of giving up on their path to parenthood.

*Ok. As a person, I am used to focus on the bright side of life… on the bright side of our attempt to become parents. I was thinking that we were capable of doing that—a possibility was there.*

(Marcella)

*Yes, we wanted two years off… We needed that, in order to calm ourselves down… mentally…*

(Calliope)

#### 3.3.3. Making Sense of Life Through the Child

Having a child through IVF gave couples meaning, while those without children found purpose in their daily lives. However, IVF failure led some to feel purposeless, causing depression and loneliness.

*As starting point, you should want children. Now, we would like to have children.*

(Menelaos)

*Our relationship with the child! How much I wanted this child! Now, our whole life revolves around them. We’re not those parents who never let anyone else look after the child and stay with them all day—no! But all our thoughts are centred around them.*

(Marcella)

#### 3.3.4. Reorienting Towards the Future After Successful IVF

Successful IVF conception brought couples a mixture of joy and anxiety. Happiness about the positive outcome helped them move beyond past struggles, while some women experienced anticipatory feelings about the pregnancy before confirming it.

*True happiness… What else?… I was suffering from anxiety in order to be sure that everything is fine… I was expecting the positive to come.*

(Hercules)

*When the doctor told us that the test was positive… I was deeply moved… I felt overwhelmed and kept wondering how the baby would finally be born.*

(Marcella)

#### 3.3.5. Commodified Hope: IVF Between Care and Market Logic

Couples interpreted IVF as financially demanding, sometimes being associated with commercial interests. They also expressed difficulty in finding the appropriate doctor and IVF centre to improve their chances of becoming parents.

*Entire business… When IVF treatment has business aspects, you are not sure about the professional’s pure intentions and job…*

(Marcella)

#### 3.3.6. Living with Uncertainty and Perceived Risks During IVF

IVF treatment sparked fears related to genetic conditions, limited information, and external risks such as COVID-19.

*There was a 25% chance it would have thalassemia, a 25% chance it would carry the trait, and a 25% chance it would have nothing at all… and another 25% chance it would carry the other trait. … We wouldn’t keep it. I wouldn’t keep it. Thalassemia—there is no straight line forward. It means a sick child we would be caring for the rest of its life. There is no cure!*

(Markella)

*Yes, we didn’t get vaccinated because we were afraid of the vaccine… to be honest… and then we caught COVID… but not at [she mentions the fertility Centre]”… we caught it at the dentist.*

(Hyacinth)

#### 3.3.7. Reconsidering Pathways to Parenthood

After repeated unsuccessful attempts, some couples began to reconsider alternative routes to parenthood, such as adoption.

*We reached a point—the most extreme point—where we said that even if we couldn’t have a child, we would adopt one.*

(Isokrates)

*We have recently submitted our application in order to adopt a child.*

(Calliope)

### 3.4. Navigating Institutional and Systemic Barriers

This theme captures couples’ perceptions of and efforts to navigate healthcare and insurance systems, including IVF centre selection, the lack of complete information about MAR and IVF, and referrals to other assisted reproduction methods.

#### 3.4.1. Negotiating Access to IVF Within Economic and Systemic Constraints

Couples selected an IVF centre based on recommendations from loved ones or gynaecologists. Their critical reflections on the healthcare system were shaped by economic barriers and limited access to IVF, with health insurance providing minimal coverage.

*It was a public hospital with an excellent IVF department, recommended by our gynaecologist…. The public healthcare and insurance system did not cover IVF treatment, requiring individuals to pay for everything out of pocket.*

(Marcella)

#### 3.4.2. Searching for Reliable Knowledge in the Absence of Adequate Information

Couples highlighted the lack of complete information about MAR and IVF and believed that institutions such as schools, universities, state agencies, and healthcare professionals should play a greater role in providing IVF information.

*There is better information about IVF now than in previous years, but it largely requires personal effort, with much information available online.*

(Marcella)

#### 3.4.3. Balancing Between Supportive Care and Social Misconceptions About IVF

Overall, couples had a positive experience at IVF centres, praising the staff’s professionalism, though they noted misconceptions and prejudices towards infertile couples and their decision to pursue IVF.

*Prejudices existed, with people assuming my sperm was unhealthy and considering us a useless, infertile couple.*

(Menelaos)

#### 3.4.4. Negotiating Moral Positions Towards Different MAR Technologies

Couples also reflected on other MAR technologies such as Preimplantation Diagnosis, Postmortem Fertilisation, Cryopreservation, and Egg Donation, expressing mixed attitudes that ranged from support to ethical hesitation.

*What I wanted to do, but I did not! I wanted to do the Preimplantation Diagnosis, in order to inspect or to fix an embryo problem prior Research to its conception.*

(Marcella)

### 3.5. Projecting the Future Though IVF Experience

This theme focuses on couples’ views of their future with children, shaped by stress and anxiety about their children’s safety and upbringing, in addition to expectations for their short- and long-term futures.

#### 3.5.1. Parenthood Under the Shadow of Age and Responsibility

New parenthood concerns regarding raising children and age-related anxiety about ensuring their well-being were raised by the participants.

*Now, yes, we worry about how we should properly grow our children.*

(Calliope)

*Well… that they will have finished their studies, that everything will be going well… and that they will start building their own lives… I don’t know… the usual dreams that every mother has for her children.*

(Thetis)

#### 3.5.2. Orienting Towards the Children’s Future

Couples’ near-future expectations were strongly oriented towards their children’s health, education, and needs. In the distant future, parental expectations centred on their children’s development and freedom to live a life of kindness and integrity.

*I hope for my child to have good health above all. In the next five years, my child will be starting school.*

(Marcella)

#### 3.5.3. Linking Personal IVF Experiences to Broader Policy Gaps and Structural Inequalities

Couples critically situated their accounts within broader public discussions about population decline in Greece and called for stronger state support for assisted reproductive services and policies addressing this issue.

*Our country has an aging population. In the next 20 years, Greece’s population will decrease to 8 million… The State should encourage couples to have children.*

(Hercules)

*The State should cover IVF treatment by 100%—through healthcare and insurance system. … [the State] should give financial funding to couples undergoing IVF.*

(Calliope)

## 4. Discussion

The findings presented herein reflect interpretative analysis of a small, idiographically examined sample in Greece, aiming to illustrate how couples made sense of infertility and IVF. Our findings revealed that couples found IVF emotionally challenging, consistent with the results of previous studies. Couples sought medical help and encountered both supportive and unsupportive behaviours from their social environment. The choice of IVF centre influenced outcomes, and several couples perceived the healthcare and insurance system as providing limited financial support for IVF treatment. Additionally, participants frequently linked their experiences to broader societal concerns, including demographic change and access to fertility treatment, and suggested that stronger state support for IVF could help address these challenges.

Relationship type and quality played a central role: couples with strong spousal support described IVF as a unifying experience, whereas others articulated fears of separation or relational breakdown. These findings are consistent with studies linking infertility and IVF to conflicts and isolation [12,47]. Infertility and IVF have been linked to these factors [47], in addition to emotional transitions, psychosocial changes [48], barriers [49], and emotions such as sadness and helplessness [50]. There is a strong need for both medical and mental health support during IVF [51]. Participants described IVF as an emotionally demanding process marked by alternating hope, uncertainty, and distress [26,52].

Similar to the results of previous studies, we found that couples feared divorce, faced economic barriers, and sought to preserve mental well-being and marital stability during IVF [53,54]. These concerns were closely tied to treatment outcomes, as positive successful IVF brought happiness and a strong parent–child bond [55], while negative outcomes caused emotional strain [56]. Within this emotional context, IVF treatment choices were influenced by psychological, socioeconomic, and time-related factors [57].

Our findings further suggest that gender, socioeconomic status, and relationship dynamics shaped how IVF was experienced. In several couples’ narratives, women described experiencing greater emotional and physical strain during treatment, particularly due to the medical procedures involved, while men framed their concerns in terms of financial pressures or their partner’s well-being, echoing previous research on gendered experiences of IVF [20,21,58,59]. Furthermore, the IVF experience affected emotional well-being and relationships, with couples’ self-reliance and psychological support being crucial [60].

Our study captured couples’ hope and their “never give up” mentality on their journey to parenthood. IVF is a process filled with stress and anxiety [38,49,61], yet it represents couples’ hope and determination to fulfil their parenthood dream, akin to climbing a mountain step by step [62].

Some participants described experiencing or perceiving prejudice related to infertility, including assumptions about reproductive health, even among IVF clinic staff, similar to the results of previous studies [63,64,65]. The findings of previous studies have confirmed that IVF conception is culturally shaped [66], and IVF clinic staff often have their own subjective perspectives on fertility, experiencing dynamic emotions within their work environment [67].

Lastly, couples’ narratives highlighted economic, healthcare, and insurance barriers that intensified distress among less affluent participants, who experienced treatment as financially draining and, at times, exploitative. These accounts underscore the need for healthcare and insurance system restructuring, combined with policies that address infertility within the context of population aging [68,69].

## 5. Strengths and Limitations

While the authors of qualitative studies on IVF in Greece have examined women’s experiences, in this study, we adopted an idiographic, dyadic IPA approach to explore the lived experiences of couples undergoing IVF, providing valuable insights into their psychological process and the support they received. From a methodological point of view, the intentional decision to interview couples and jointly analyse partners’ narratives enabled us to explore the relational dynamics among couples. However, consistent with all qualitative studies, the small, non-representative sample limits generalisability. Additionally, contextual constraints on data collection—such as conducting some interviews in semi-public settings (e.g., cafés)—may have shaped the depth or disclosure of participants’ accounts. The sensitive nature of the research topic, which involved personal and family life (privacy concerns), limited the diversity of perspectives. Lastly, data saturation, in the traditional sense, was not achieved, yet this decision was intentional and consistent with the IPA approach, which prioritises depth of understanding over breadth. As a result, in our study, the six participating couples provided sufficiently rich and detailed narratives to address the research questions and generate meaningful insights.

## 6. Implications and Recommendations for Future Research

The findings of this study have clinical and policy implications. First, psychological care for couples provided by experienced and qualified psychologists could support couples’ sense-making, reduce treatment-related distress, and strengthen dyadic coping during IVF cycles, aligning with relevant recommendations [70]. Second, participants’ call for state support could take the form of public and private sector partnerships to co-finance IVF or ART, a model used internationally [68], and address Greece’s population decline. Lastly, the authors of future studies could explore attitudes towards ART among infertile couples and the general public, compare the experiences of women undergoing IVF with those using alternative ART methods, and evaluate the degree to which financing arrangements and integrated psychosocial care contribute to treatment engagement and well-being.

## 7. Conclusions

The findings presented herein highlight the emotional challenges, social pressures, and attitudes towards IVF and assisted reproductive methods. Despite improved perceptions, gaps in information and stereotypes continue to affect infertile couples pursuing parenthood through IVF.

## Figures and Tables

**Table 1:** Demographic characteristics of the participating couples.

Pseudonym	Age		Years Undergone IVF		Residence	Already Had a Child
Thetis and Hercules	48 and 49		2 years		Karditsa	Yes (with IVF)
Aelia and Herodotus	40 and 48		5 years		Athens	Yes (naturally)
Calliope and Menelaos	47		7 years		Athens	No
Hyacinth and Archelaus	41 and 49		8 years		Athens	No
Marcella and Isokrates	40 and 48		2 years		Karditsa	Yes (with IVF)
Julia and Aristeidis	40 and 43		10 years		Karditsa	Yes (naturally)
	Mean	Range	Mean	Range		
All couples	44.33	40–49	5.66	1–10		

## Data Availability

Selected excerpts from participants’ original contributions in this study are included in the article as interview quotations. Further enquiries can be directed to the corresponding author. Qualitative data cannot be made publicly available due to the sensitive and potentially identifiable nature of the information provided by participants. Even after anonymisation, full transcripts may contain contextual details that could compromise participant confidentiality. To protect participants’ privacy and comply with ethical approvals and informed consent agreements, the data are therefore available from the authors upon reasonable request.

## Supplementary Materials

The following supporting information can be downloaded at https://www.mdpi.com/article/10.3390/healthcare14060802/s1: Document S1: Interview Guide; Table S1: COREQ: 32-item checklist [45]; Table S2: Superordinate and subordinate themes.

## Abbreviations

The following abbreviations are used in this manuscript:

IVF	In Vitro Fertilisation
ART	Assisted Reproductive Technology
IPA	Interpretative Phenomenological Analysis
