# Supplementary material for: The Lived Experience of Couples Undergoing In Vitro Fertilisation in Greece: An Interpretative Phenomenological Analysis
Source: Healthcare (Basel). 2026 Mar 21;14(6):802. doi: 10.3390/healthcare14060802 (PMC13027371; doi:10.3390/healthcare14060802)
Supplement: Supplementary file 1 [file healthcare-14-00802-s001.zip › IVF Supplementary document S1_Interview Guide R.pdf]

The lived experience of couples undergoing in vitro fertilisation in Greece: An Interpretative Phenomenological Analysis

Interview Guide

- **Experiences before undergoing IVF**

1. How did you made the decision to undergo *in vitro* fertilisation treatment as a couple?
   a. How did you feel by taking this decision?
   b. What kind of thoughts were on your minds, during the process of your decision-making?
2. Did you share your decision with other people?
   a. What kind of other people's attitudes had you observed towards in-vitro fertilisation treatment and you- as couple and as future parents through in-vitro fertilisation treatment-by sharing that decision?
   b. What kind of other people's reactions did you observe, through this kind of decision sharing?
3. What kind of information do you have about Medically Assisted Reproduction and *in vitro* fertilisation treatment?
4. What did *in vitro* fertilisation treatment mean to you?

- **The IVF process**

1.Could you share with me, some information, about the *in vitro* fertilisation treatment's procedure?

a. How did you choose the specific doctor/ *in vitro* fertilisation treatment's Center?

b. How did you feel?

c. How was your interaction with the medical staff?

d. How was your interaction with the Healthcare/Insurance System?

e. (If *in vitro* fertilisation treatment took place during COVID-19 era): How did pandemic era influence your *in vitro* fertilisation treatment?

2. What was your *in vitro* fertilisation treatment's result?

a. How do you feel about the result?

b. (If *in vitro* fertilisation treatment was unsuccessful): Do you think to try again?

Life after IVF

1.How is your life today, after *in vitro* fertilisation treatment?

a. How are you as a couple, today?

b. How is your relationship with your child (if *in vitro* fertilisation treatment was successful)

c. Did you share or are you going to share with your child, that he/she came through *in vitro* fertilisation treatment?

2. How do you imagine your life in the upcoming five years, after this experience?

a. After twenty years?

3.What kind of role, did you believe, that State has through its Healthcare System according Medically Assisted Reproduction?

a. Do you believe, that exists the appropriate information, according Medically Assisted Reproduction nowadays?

b. Do you believe, that exists the appropriate insurance coverage towards Medically Assisted Reproduction?

4. What do you believe about Greek people's attitude towards *in vitro* fertilisation treatment?
