# Supplementary material for: The Lived Experience of Couples Undergoing In Vitro Fertilisation in Greece: An Interpretative Phenomenological Analysis
Source: Healthcare (Basel). 2026 Mar 21;14(6):802. doi: 10.3390/healthcare14060802 (PMC13027371; doi:10.3390/healthcare14060802)
Supplement: Supplementary file 1 [file healthcare-14-00802-s001.zip › IVF Supplementary Table S2_THEMES R.pdf]

The lived experience of couples undergoing in vitro fertilisation in Greece: An interpretative Phenomenological Analysis

Table S2. Superordinate and subordinate themes.

**Superordinate theme**	**Subordinate themes**
Making sense of infertility and IVF	Experiencing infertility<br>Seeking help for IVF<br>Making sense of IVF
Negotiating relationships under the strain of IVF	Strengthening spousal and parental bond<br>Negotiating disclosure and social reactions to IVF
IVF as an emotionally demanding journey	Enduring the emotional and physical challenges of IVF<br>Resilience and Imposter Syndrome<br>Making sense of life through the child<br>Reorienting towards the future after successful IVF<br>Commodified hope: IVF between care and market logic<br>Living with uncertainty and perceived risks during IVF<br>Reconsidering pathways to parenthood
Navigating institutional and systemic barriers	Negotiating access to IVF within economic and systemic constraints<br>Searching for reliable knowledge in the absence of adequate information<br>Balancing between supportive care and social misconceptions about IVF<br>Negotiating moral positions towards different MAR technologies
Projecting the future though IVF experience	Parenthood under the shadow of age and responsibility<br>Orienting towards the children’s future<br>Linking personal IVF experiences to broader policy gaps and structural inequalities
